# Disentangling the Diversity of Arboreal Ant Communities in Tropical Forest Trees

**DOI:** 10.1371/journal.pone.0117853

**Published:** 2015-02-25

**Authors:** Petr Klimes, Pavel Fibich, Cliffson Idigel, Maling Rimandai

**Affiliations:** 1 Institute of Entomology, Biology Centre of the Czech Academy of Sciences, Ceske Budejovice, Czech Republic; 2 Faculty of Science, University of South Bohemia, Ceske Budejovice, Czech Republic; 3 New Guinea Binatang Research Center, Madang, Papua New Guinea; Universidade Federal de Goiás, BRAZIL

## Abstract

Tropical canopies are known for their high abundance and diversity of ants. However, the factors which enable coexistence of so many species in trees, and in particular, the role of foragers in determining local diversity, are not well understood. We censused nesting and foraging arboreal ant communities in two 0.32 ha plots of primary and secondary lowland rainforest in New Guinea and explored their species diversity and composition. Null models were used to test if the records of species foraging (but not nesting) in a tree were dependent on the spatial distribution of nests in surrounding trees. In total, 102 ant species from 389 trees occurred in the primary plot compared with only 50 species from 295 trees in the secondary forest plot. However, there was only a small difference in mean ant richness per tree between primary and secondary forest (3.8 and 3.3 sp. respectively) and considerably lower richness per tree was found only when nests were considered (1.5 sp. in both forests). About half of foraging individuals collected in a tree belonged to species which were not nesting in that tree. Null models showed that the ants foraging but not nesting in a tree are more likely to nest in nearby trees than would be expected at random. The effects of both forest stage and tree size traits were similar regardless of whether only foragers, only nests, or both datasets combined were considered. However, relative abundance distributions of species differed between foraging and nesting communities. The primary forest plot was dominated by native ant species, whereas invasive species were common in secondary forest. This study demonstrates the high contribution of foragers to arboreal ant diversity, indicating an important role of connectivity between trees, and also highlights the importance of primary vegetation for the conservation of native ant communities.

## Introduction

Tropical forests play a prominent role in the maintenance of global biodiversity and ecosystem processes [1,2]. Their canopies are known to support an incredible diversity of animal species, in particular that of arthropods [3,4]. Despite growing interest in canopy research, we still know relatively little about the ecology and biology of arboreal arthropod fauna and the processes that maintain its diversity and distribution [1,3,5]. Such knowledge is crucial as tropical forests are increasingly logged and cleared to become structurally simpler environments such as secondary forests and plantations [2,6–8]. These altered ecosystems usually have a lower diversity both of animals and plants, simpler vegetation structure, and altered species composition. However, the effects of forest disturbance and fragmentation can also differ markedly between animal taxa [9–11] making policy decisions and ecological predictions without deeper knowledge on the ecology of different groups difficult.

Ants are one of the most diverse, abundant and ecologically important animals in tropical forest ecosystems [12,13]. An extraordinary abundance of ants is typical for rainforest canopies, where ants represent 20–60% of the total arthropod biomass and up to 90% of individuals in samples obtained by insecticidal fogging [14–16]. A good taxonomic knowledge base and sensitivity to environmental changes make ants a suitable animal group for biodiversity and ecological studies of tropical forest fauna [17,18]. In particular, ants are regarded together with birds and butterflies as suitable indicators of rainforest disturbance [9,10,17,19].

Positive and negative interactions between species, territoriality and competition are considered to be the major mechanisms structuring arboreal ant communities [16,18]. However, other biotic and abiotic factors such as nest-site availability and vegetation structure [20–22], plant diversity [23,24], habitat disturbance [25,26] and distribution of food resources [27,28] have also been shown more recently to be important. Despite this knowledge, it is still disputed whether ant community assembly processes in rainforests are determined by environmental factors, species interactions, or rather are structured randomly [28–32]. In particular, we still know very little about how interactions between species and their nesting resources (i.e. trees) influence the coexistence of arboreal ant species at small spatial scales [20,33]. Moreover, most of our knowledge about distribution of their communities in tropical trees is based on data from disturbed habitats, such as plantations and secondary forests, with much lower plant diversity [21,34–37], or from isolated trees [38], whilst similar studies from primary forests conducted on the level of whole communities of plants and ants are relatively scarce; but see [22,39]. In particular, most of existing studies of undisturbed tropical forests and savannas are limited to lower canopy strata or to a small number of trees, e.g. [20,26,29,33,40,41], or are focused only on the most common (dominant) ant species, e.g. [42–44].

Although arboreal ant communities in tropical forests can be species-rich, they are usually less diverse in their taxonomic composition than the leaf-litter fauna [17,45]. However, it has recently been estimated that almost half of all ant species in tropical forests might be at least partly associated with tree canopies [46] and over 40 species have been recorded on a single tropical tree [29,47]. Despite this high diversity, arboreal ant communities are typically dominated (numerically and behaviorally) by just one to three common species, with the remaining species being much rarer [29,35,43]. It is not yet clear how species that vary so much in abundance can coexist in a tree. In particular, even the elemental question of what proportion of ant species present on a tree nest there and what proportion are only visitors from other trees (or forest floor) remains unknown.

Because tree canopies are challenging to access, most previous studies of canopy ants have used indirect methods, such as fogging and various canopy traps, rather than direct sampling [15,20,48]. Although there have recently been attempts to improve methods for the rapid assessment of arboreal arthropod communities using multiple sampling techniques [49,50], such surveys usually do not obtain detailed data about the biology and ecology of the species. The current figures regarding the ant diversity supported by individual trees may also be biased as these studies fail to distinguish foragers from nearby trees and leaf-litter from the fauna that actually nest in that tree [25,51]. To our knowledge, there are only two studies of rainforest arboreal ants that explored not only their species total diversity but also activity (i.e. foraging at baits and on tree) and searched for presence of nests in individual trees [33,51]. Nevertheless, these studies did not attempt to compare the diversity and composition of complete communities of ant foragers and arboreal nests. Moreover, there has been no quantitative comparison of primary and secondary forest whole communities from continuous patches of forest. We argue that such intensive studies based on plot-scale inventories of whole assemblages are needed to assess the diversity, composition, foraging and nesting frequency of ant communities in trees, and the manner in which they respond to forest disturbance and tree traits.

Here, we sampled the complete ant fauna in trees in one plot of an old-growth (primary) and one plot of a secondary lowland rainforest in Papua New Guinea (PNG). A previous study on those plots demonstrated that successional determinants and differences in species turnover (beta diversity) are responsible for the higher number of species nesting in trees in primary than in secondary forests [22] and was limited solely to ant nests and species densities. In contrast, in this study we focus on the overall diversity and composition of entire arboreal communities (including foragers) in whole plots and in individual trees (gamma and alpha species diversity respectively) and assess the effects of forest successional stage and tree size traits on species composition. For the first time, we compare tree-nesting to foraging communities, which enables us to distinguish what proportion of the ant species are solely foraging in individual trees. We then explore whether foraging ants in a tree are more likely to nest in surrounding trees than would be expected by random. We hypothesize that the species diversity of both ant foragers and entire communities will be higher in the primary forest than in secondary forest at both the individual tree and whole-plot levels [22,52]. Furthermore, we expect that ant communities in the primary rainforest plot will be more distinct in their taxonomic composition and will be characterized by the presence of native ant species, whilst opportunistic and invasive species will be more common in the secondary forest plot due its recent disturbance [32, 53]. Finally, we predict that a high proportion of ant diversity in individual trees can be attributed to foragers from surrounding vegetation [51,54], indicating that foraging diversity might be spatially dependent.

## Materials and Methods

### Ethics Statement

The research has been conducted according to the PNG law. All work in the field has been conducted in close collaboration with PNG customary landowners of Wanang (05°14′S 145°11′E) in their forests and with their permission, who are the exclusive private owners of the land (community leader Mr. Filip Damen should be contacted for future permissions). No specific permit was needed for the specimen collection because none of the insect species studied here is protected. Permits to export the collected insect samples from PNG to Institute of Entomology of Biology Centre ASCR (Czech Republic) were provided by the Department of Environment and Conservation, Boroko, National Capital District, PNG (permit No: 070382).

### Study site and characteristic of the forest plots

The study site was located in a rainforest inland area near Wanang village, Madang province, PNG (100–200 m. a. s. l.), a small village with population ~200 surrounded by extensive areas of lowland forest and a locally managed conservation area of 10,770 ha [55].The area is part of an extensive evergreen rainforest ecosystem on latosols in the basin of the Ramu river [56], partly used for slash-and-burn agriculture at its margins. The climate in the region is perhumid with mean annual rainfall of 3,500 mm and a mild dry season from July to September; mean air temperature is 26.5°C, which varies little throughout the year [57].

Arboreal arthropods and trees were intensively sampled in two one-hectare quadrat plots, which were approximately one km from each other [58,59]. Here we focus on arboreal ant communities studied in 0.32 ha of each 1-ha forest plot (40 m × 80 m section), where comparable data on censuses of both ant foragers and nesters were collected. In total, 684 trees with diameter at breast height (DBH) ≥ 5 cm were sampled. One plot was located in a small area of secondary forest surrounded by pristine forests (about 10 years old succession on an abandoned garden of size of several ha and canopy height of 25 m) and the second plot in a primary forest with canopy height up to 50 m (i.e. mature forest with no disturbance for over 50 years) [59,60]. Tree communities in the primary forest plot were more than twice as diverse as those in secondary forest plot (115 and 47 species respectively), with the most common genera *Horsfieldia* (Myristicaceae), *Teijsmanniodendron* (Lamiaceae) and *Gymnacranthera* (Myristicaceae). The most common genera in the secondary plot were *Ficus* (Moraceae), *Trichospermum* (Malvaceae) and *Macaranga* (Euphorbiaceae) (for more vegetation characteristics see Table 1 and [59,60]). The two plots were selected in cooperation with indigenous landowners who practice swidden agriculture at the site to cultivate traditional crops. This partnership allowed researchers to intensively sample trees felled by villagers, while not contributing to further deforestation and to simultaneously support the conservation of the surrounding rainforests [59,61].

**Table 1:** Characteristics of vegetation in 0.32 ha plots of primary and secondary rainforest (all trees with DBH ≥ 5 cm).

Forest plots (area)	n of stems	Basal area (m^2^)	Tree height per tree (mean and min—max, m)	DBH per tree (mean and min—max, cm)	Leaf weight per tree (mean and min—max, kg)	n of sp.	n of genera	n of families
**Primary (0.32 ha)**	389	8.97	14.2 (4.6–51.8)	12.7 (5.0–99.8)	8.3 (0.1–105.5)	115	78	41
**Secondary (0.32 ha)**	295	3.88	12.5 (3.2–24.0)	11.3 (5.0–43.5)	6.5 (0.1–68.4)	47	33	19
**Both plots combined (0.64 ha)**	684	12.85	13.5 (3.2–51.8)	12.1 (5.0–99.8)	7.5 (0.1–105.5)	145	98	43

### Sampling design and material collection

Each forest plot was extensively sampled for ants using a standardized protocol (i.e. equal sampling effort per tree in both plots). All trees with DBH ≥ 5 cm were marked and then felled. In total, ants from 389 trees in the primary plot and from 295 trees in the secondary plot were sampled and their spatial position (coordinates of trunks) within the plot measured. The sampling continued for 10 months, from February to November 2007, with an average of 5.6 trees examined per day (range: 1–12 trees). There was no significant effect of sampling date on ant diversity per tree in either primary forest (linear regression of number of species with date; F_1,387_ = 0.26, p = 0.6, R^2^ ≤ 0.001) or secondary forest (F_1,293_ = 1.1, p = 0.3, R^2^ = 0.004). Furthermore, three 0.1 ha subsections in each plot varied only a little in ant nest diversity and density in trees [22]. DBH (cm), trunk height (m), crown width and crown height (m) and total fresh leaf weight for each tree (kg) were measured [59] (referred to as tree traits hereafter). Every tree was intensively searched when felled for nests and foraging individuals for between 10 and 120 min (census time adjusted according to tree size) by a team of three collectors. One collector sampled for ant foragers along the whole tree from trunk base to crown and two collectors searched specifically for nests. This method allowed for the cutting of all branches, attached lianas and the dissection of parts of the trunk, bark, and inspection of epiphytic aerial soil, which permitted the recording of virtually the entire ant fauna of each tree. All attached lianas and epiphytes were also sampled in each tree and analyzed as components of their host trees (including woody climbers with DBH ≥ 5 cm).

Because all trees in the plot were not felled simultaneously we did not attempt to experimentally determine colony boundaries; instead only ant nests were counted. Nests were defined as (i) spatially segregated microhabitats (cavities) in trees where workers and queen(s) and/or immature life stages were found or (ii) ant-build carton, silk and soil formations on the bark and on leaves (including satellite nests with workers only). All other species occurrences in a tree were considered as foragers. If it was not possible to discern separate nests of the same ant species within one tree and same microhabitat (i.e. continuous habitat as e.g. moss cover, twigs and branches), only nest chambers separated from each other by vertical distance > 1 m were treated as separate records. Samples of several individuals from each nest (including all observed castes) and a pooled sample of ant foragers collected per tree (typically one to several hundred individuals) were stored in vials with absolute ethanol for later identification in the laboratory.

### Species identification

All ants were first sorted to caste (minor workers, major workers, males, queens), morphotyped, and identified to genus using Bolton [62]. Morphospecies were determined to species using reference collections at the Biology Centre of the Czech Academy of Sciences, Australian National Insect Collections (CSIRO) and Harvard Museum of Natural History, online image databases (www.antweb.org; www.newguineants.org), and with the additional assistance of taxonomists (see acknowledgments). Most of the ant species were barcoded for COI sequences, with the exception of a small number of rare taxa. Morphological identifications and obtained sequences were then compared with COI sequences and photos in the online database of the Consortium for the Barcoding of Life (www.formicidaebol.org). The combination of morphological (typically including all castes from a nest series) and molecular analysis helped to define species concepts of the poorly known ant fauna. Vouchers of ant species are deposited at the Institute of Entomology, Biology Centre of Czech Academy of Sciences. Species codes are used for sake of clarity in the figures hereafter (i.e. the first four letters of the genus and three digits) and full species names are available in S1 Table. Taxa names were updated according the current changes in ant nomenclature [63].

### Data Analysis

#### Note on replication

Our analysis focused on ant communities from individual trees, which constitute replicated data points within each primary and secondary forest plot. However, the plots themselves could not be replicated due to the logistical demands and costs to fell and survey all trees with DBH ≥ 5 cm. Our data used for the comparison between primary and secondary forest are thus pseudoreplicated [64]. This is a common limit for studies at the level of whole ecosystems and for those focusing on superabundant and diverse taxa like insects. It has been argued that in such cases a cautious use of inferential statistic is acceptable, especially where a larger scale has priority over replication [65]. The statistical methods used here should be viewed as tests for differences between our plots rather than a generalized comparison of the two distinct forest types. Nevertheless, additional vegetation data from the study site show that our chosen plots reflect the typical vegetation structure and diversity of primary and secondary lowland forests of PNG [60,66].

#### Ant diversity and abundance

Sample-based species accumulation curves were calculated to explore the relationship between species richness and number of sampled trees in each forest plot with trees regarded as samples. The Mao Tau function in EstimateS version 8.2 was used for calculations [67]. The curves were first produced separately using datasets on nesting and foraging communities (S2 Table), and then for both datasets combined. The overall ant diversity within each forest plot was estimated using Chao2 based on 100 randomizations [67]. The mean ant species richness per tree was also calculated separately for nesting and foraging species, and for whole fauna combined, and compared between forest plots using one-way ANOVA. The mean number of foraging individuals and number of nests per tree (as measurements of ant activity and abundance) was compared between the forest plots for all of the above combinations. Additionally, we compared the mean diversities and abundances of ant species that foraged and did not nest on a tree, but nested in other trees in the plot to assess the relative proportion of foraging arboreal “tourist species” per tree (i.e. foraging minus nesting; F-N hereafter, Table 2). The abundances of ants, nests and species were log-transformed prior to analyses to achieve data normality and homoscedasticity. Frequencies of ant species recorded as nesting, foraging or both were compared between forest plots using contingency tables with maximum-likelihood chi-square tests. Univariate analyses were performed in STATISTICA ver. 9.1 [68].

**Table 2:** Characteristics of arboreal ant communities in 0.32 ha plots of primary and secondary rainforest (in trees with DBH ≥ 5 cm).

Forest	Overall ant species richness (n of species)				All species per tree ± SE	Nesting (N) ants per tree ± SE		Foraging (F) ants per tree ± SE		F-N ants per tree ± SE	
(plot)	All	N	F	F-N	n of species	n of nests	n of species	n of coll. individuals	n of species	n of coll. individuals	n of species
**Primary**	102	80	72	45	3.8 ± 0.1^**A**^ (max. 20)	1.9 ± 0.1^**A**^ (max. 20)	1.5 ± 0.1^**A**^ (max. 12)	45.5 ± 2.9^**A**^ (max. 320)	2.9 ± 0.1^**A**^ (max. 12)	25.5 ± 1.9^**A**^ (max. 291)	2.2 ± 0.1^**A**^ (max. 9)
**Secondary**	50	42	38	28	3.3 ± 0.1^**B**^ (max. 14)	2.0 ± 0.1^**A**^ (max. 13)	1.5 ± 0.1^**A**^ (max. 8)	16.7 ± 1.2^**B**^ (max. 111)	2.3 ± 0.1^**B**^ (max. 9)	9.9 ± 0.8^**B**^ (max. 88)	1.7 ± 0.1^**B**^ (max. 9)
**Both plots combined**	126	99	96	64	3.6 ± 0.1 (max. 20)	1.9 ± 0.1 (max. 20)	1.5 ± 0.1 (max. 12)	33.1 ± 1.8 (max. 320)	2.7 ± 0.1 (max. 12)	18.8 ± 1.2 (max. 291)	2.0 ± 0.1 (max. 9)

Different capital letters within each column indicate a significant difference between primary and secondary forest plots (mean ± SE, ANOVA on log-transformed data, *P* < 0.05). F-N indicates values for arboricolous ant species that foraged and did not nest on tree (species with ≥ 1 nest per plot included).

#### Composition of ant communities

Multivariate ordination analyses were carried out to assess the relationship between ant communities and the measured environmental variables using CANOCO Version 4.5 [69]. We aimed to compare the effects of tested variables on ant species composition between the different surveys for i) all ant occurrences combined, ii) foraging occurrences and iii) arboreal-nesting occurrences. To achieve this, presence-absence matrices of species as columns and sampled trees as rows were prepared and analyzed for each of the datasets separately (S2 Table). Unimodal canonical methods were chosen because of the relatively high species turnover (axis length > 4.0 in detrended canonical analyses) and because our main aim was to test the relative differences in species composition between the two plots [70]. First, canonical unconstrained analysis (CA) was performed. Canonical correspondence analysis (CCA) was then used to test for the effect of forest plot (primary and secondary) and tree traits on ant species composition (Table 3). Because we assumed that all tree traits were correlated (i.e. related to tree size), all traits were first included in the analysis and forward selection was applied to test which variables had significant influences on species composition [70]. All trees and ant species were included and the environmental variables were log-transformed prior to analyses. The option of ‘down-weighting of rare species’ in Canoco was applied to decrease the influence of rare ant taxa. The environmental variables (ordination axes) correlated with species composition were then tested for significance using Monte-Carlo permutations (F ratio, P < 0.05, 999 runs per analysis). The percentage of the variability effectively explained by the tested variables in species data was expressed as the proportion of total variability on each of canonical axis (% ratio of CCA to CA axes; Table 3). An ordination diagram based on inter-sample distances was constructed for all ant records to illustrate the differences in overall species composition between primary and secondary forest.

**Table 3:** Multivariate analyses of the effect of forest plot (primary and secondary) and tree size traits (DBH, tree height, trunk height, crown height, crown width, total leaves weight; log-transformed data) on ant species composition.

Dataset and canonical axes^**a**^	CA Eigenvalue^**b**^	CA %^**c**^	CCA Eigenvalue	Variable	CCA %^c^	F^**d**^	p	(CCA/CA) %^**e**^
**All records (647 trees × 126 species)**								
**All axes**	12.88	100	0.97	All variables	7.5	7.4	0.001	7.5
**1**	1.00	7.8	0.81	Forest plot	6.3	43.1	0.001	80.8
**2**	0.85	6.5	0.07	log(DBH)	0.5	3.9	0.001	7.7
**Foraging (561 trees × 96 species)**								
**All axes**	8.65	100	0.98	All variables	11.3	10.1	0.001	11.3
**1**	1.00	11.6	0.86	Forest plot	9.9	61.4	0.001	85.3
**2**	0.89	10.2	0.04	log(DBH)	0.5	2.6	0.003	4.9
**3**	0.48	5.5	0.02	log(Tree height)	0.2	1.1	0.025	3.6
**4**	0.41	4.7	0.02	log(Crown width)	0.2	1.6	0.035	4.3
**Nesting (497 trees × 99 species)**								
**All axes**	30.76	100	1.17	All variables	3.8	2.8	0.001	3.8
**1**	1.00	3.3	0.76	Forest plot	2.5	12.5	0.001	75.8
**2**	0.89	2.8	0.19	log(DBH)	0.6	3.2	0.001	21.4

Canonical analysis (CA) and canonical correspondence analysis (CCA) was performed on ant presence-absence data (i.e. number of occupied trees by species) for i) all ant occurrences combined, ii) foraging ant occurrences and iii) arboreal-nesting occurrences. Results are shown for all canonical axes (i.e. all variables) combined and then for each variable tested separately in order of its significance in forward selection (only significant results up to fourth canonical axis shown).

^a^Rank of canonical axis and significant variable in forward selection.

^b^Canonical eigenvalue for all axes (total value) and for each axis and corresponding variable separately.

^c^The per cent variance in species data explained by respective CA and CCA canonical axes.

^d^Significances of canonical axes assessed via Monte-Carlo permutation (F-ratio value, P < 0.05, 999 runs per analysis).

^e^The per cent variance in species data explained by variables across all and by individual CA axes.

Using the presence-absence data of ants in trees, we explored the relative distribution of species from different ant subfamilies and genera, and the distribution of the most common species in the studied plots. Additionally, abundances of foraging individuals and nests were compared graphically within and between forests for proportions of the five most common species as a relative measurement of ant abundance, and the frequencies of invasive species were marked differently from the rest of the ant fauna. The invasive species were identified according to the literature [71–73] as (i) species of exotic origin in the Papuan (Melanesian/Pacific) region or (ii) tramp species known to be abundant in disturbed habitats and reported to be invasive elsewhere (i.e. of unknown origin) (S1 Table).

#### Models on nesting probabilities of tree-foraging ant communities

A null modelling approach was used to test if records of ant species foraging (but not nesting) in a tree were dependent on the spatial distribution of nests of that species in surrounding trees [74]. Presence-absence data of ant species in terms of both their foraging and nesting records were used for all spatial analyses (S2 Table). Distance between tree trunks was used as a surrogate for distances between nests. For each tree and each F-N species we computed a distance to the nearest tree with a nest of that species. A cumulative probability curve of the nearest distances to the nests was then constructed using the matrix of distances across F-N species (S3 Table). Furthermore, the mean probability of nest occurrence of F-N species in other trees was calculated as the proportion of the number of observed nests from the total number of potential nests (i.e. all trees) occurring up to maximum distance *d*. The probability value was evaluated for each tree ant community (i.e. per F-N species) and increasing *d* = 5, 10, 15,…, 30 m (see S1 Text for details on calculation). The model thus took into account the number of trees up to the given distance (i.e. tree density) as well as the number of different F-N ant species on each focal tree (see S4 Table for resulted probabilities). All F-N species were considered except *Anoplolepis gracilipes* (ANOP001), which was excluded from the spatial analyses due to its ground-level-nesting habit (i.e. presence of a single super-colony of the species covering the ground of the whole secondary forest plot with 124 trees visited by foragers, but only 4 small satellite nests found in trees; S1 Table). Trunk-foraging behavior from the forest floor to canopies is well known for the species [75,76] and was also observed here.

To distinguish observed patterns from those expected at random, 100 independent permutations of the whole input matrix of nests by trees were conducted for both cumulative probabilities of F-N species to their nearest nest and for their nesting probabilities with distance. In the permutations, each ant species and each tree had the same number of nests as in the observed plots (i.e. row and column sums of the matrix were held constant). We used this restricted permutation in both models to reflect the natural probability of ants to use each tree as a nesting resource and to account for effects of tree size, as there is a high correlation between the diversity of ants and this variable, and a relatively high proportion of small-sized trees lack nests in our plots [22]. If the observed frequency of nests is higher or lower than their frequency expected at random within a distance, then F-N ants are more aggregated (clumped) to the nests or more regularly (equally) distributed, respectively, than expected if nests are distributed in trees at random [74]. Mean distances to the nearest nest of F-N species was compared statistically to the null model distribution using Mann-Whitney-Wilcox tests; the observed nesting probabilities within a distance were considered significantly different if the mean was not overlapped by the 95% confidence intervals (2.5%–97.5% quantile range) generated by the null model. All analyses were performed in R 3.1.0 [77] using the package “vegan” version 2.0–10 [78]. The random permutations were applied using the “quasiswap” method which holds marginal totals constant and produces matrices independent from previous matrices.

## Results

### Ant diversity and abundance

Arboreal ant communities were species-rich given the rather small area of rainforest (0.64 ha) surveyed. In total, 126 ant species were collected from 684 trees. When the foraging and nesting fauna are considered separately, 22,621 individuals from 96 foraging species and 1,332 nests from 99 species were collected in total (for a full species list and their occurrences see S1 Table). Overall abundance of foraging ants and ant nests was higher in the primary than in the secondary forest plot (752 nests and 17,688 foragers versus 580 nests and 4,933 foragers). No ants were found on 4.6% and 6.4% of trees in the primary and secondary forest plots respectively. These were usually small trees with DBH less than 10 cm. Nests were found on 70% of trees and foragers were observed on 87% of trees in primary forest. Similar occupancy rates were observed for the secondary forest plot (77% of trees with nests and 74% with foragers).

**Fig 1:**
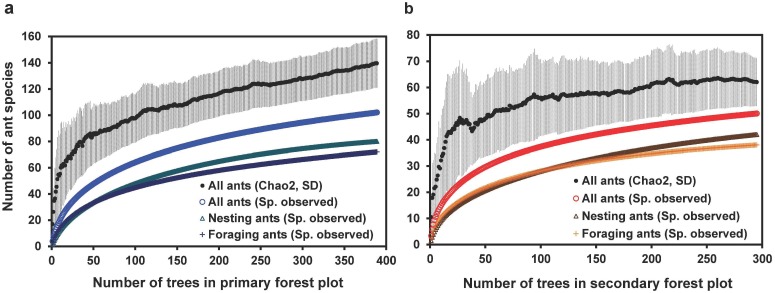
Sample-based rarefaction curves of the number of ant species in trees. Ant species richness (a) in 0.32 ha plot of primary and (b) in 0.32 ha plot of secondary rainforest. The curves for the numbers of species observed are shown separately for ant nests, foraging ant species and for both groups combined respectively. Overall diversity of ants in each forest plot is estimated using Chao 2 (mean ± SD).

The primary forest plot was approximately twice as species rich as the secondary forest plot with 102 ant species collected from 389 trees and 50 species from 295 trees respectively. The relative difference in total observed ant diversity between the two forests was similar for foraging and nesting ant species richness and species accumulation curves for nesting and foraging ants almost overlapped in both plots (Fig. 1, Table 2). However, the accumulation curves for all species records combined yielded higher values of species richness (Fig. 1). This was due to the fact that 20–30% of species were present in only one class (i.e. either as nesters or foragers), while approximately half of the species were observed to be both foraging and nesting in the studied plots (S1 Fig.). The overall estimated arboreal ant species richness was 139.5 (Chao2; SD = 18.5) species in primary forest and 62.0 (Chao2; SD = 9.2) species in secondary forest. However, the estimated species curve reached an asymptote only in secondary forest (Fig. 1).

**Fig 2:**
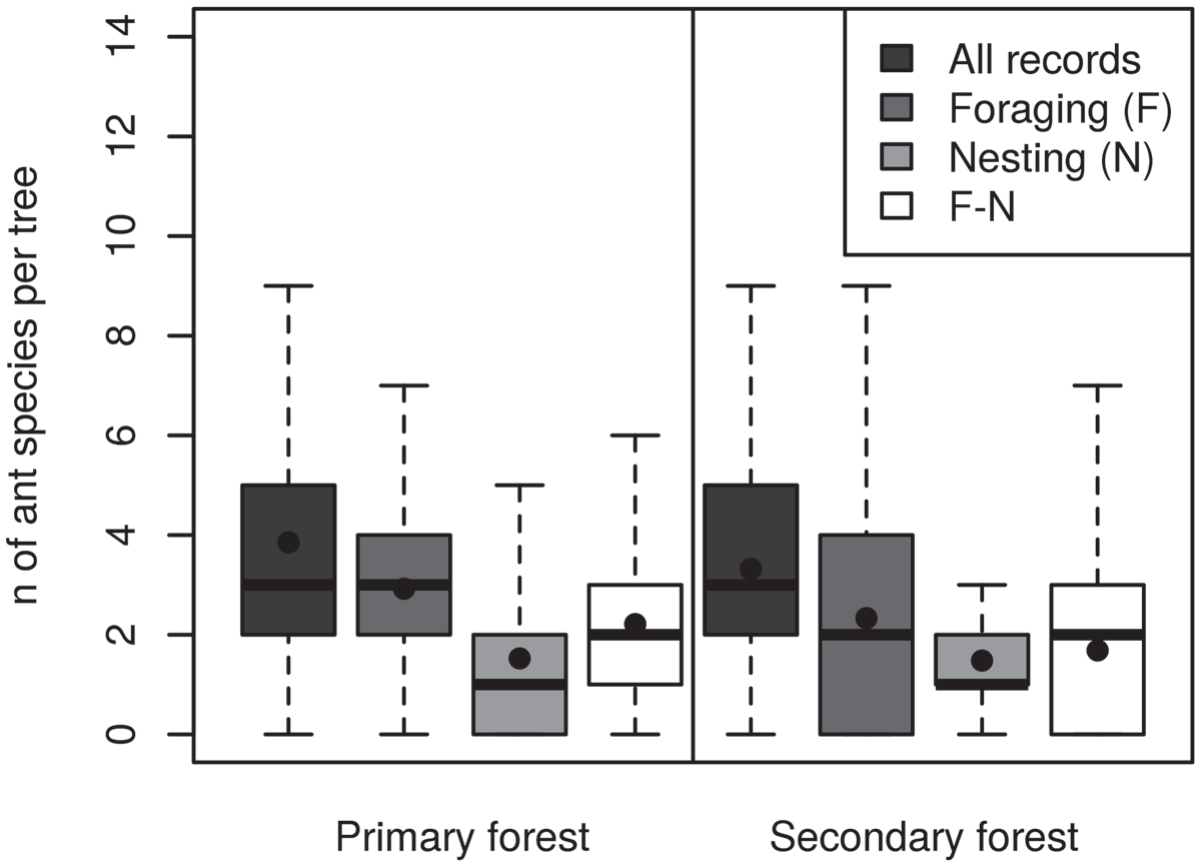
Number of ant species per tree in primary and secondary forest plot. Box-plots show median and mean values per a tree (black line and dot respectively) with 25–75% quartiles and whiskers represent 1.5 interquartile ranges for all species combined (All records), foraging species (F), nesting ant species (N) and species that foraged but did not nest on tree (F-N). Average species richness per tree is significantly higher for F than for N ants in both habitat types (paired t-test, log-transformed data, *P* < 0.001). See also Table 2 for the mean and SE values.

Mean ant species richness per tree was 3.6. However, diversity ranged widely among trees from zero species to a maximum of 20 and 14 species per tree in primary and secondary forest respectively (Table 2). The average richness per tree for all ants was slightly (but significantly) higher in primary than secondary forest trees (3.8 and 3.3 species per tree, Fig. 2, Table 2). Similarly, there were significantly fewer foraging individuals and foraging species on secondary forest trees on average. However, the mean nesting species richness and nest abundance per tree did not differ between the two forest types, with only 1.5 species and 2.0 nests recorded on average per tree in both primary and secondary forest (Table 2). In contrast, significantly more species than in nests were recorded if only foragers were considered in both forests studied (mean per tree: 2.9 species in the primary and 2.3 in the secondary forest plot). Moreover, the diversity of foragers was still higher than that for nests even if only F-N species richness per tree was considered (Fig. 2). On average, in both forest types, approximately half of the foraging individuals collected in a tree belonged to species which were not found nesting in that tree (Table 2).

### Composition of ant communities

The 126 ant species sampled belong to seven subfamilies and 37 genera. Representation of subfamilies was very similar in primary and secondary forest with ≥ 50% of all species occurrence records belonging to Formicinae, followed by Myrmicinae with ≥ 25% (S2 Fig.). No individuals representing the subfamilies Ponerinae, Ectatomminae and Dorylinae were recorded in trees in the secondary forest plot. However, these subfamilies were also poorly represented in the primary forest community (≤ 5% of occurrences). The most common genus in both forest types was *Camponotus*. The most species-rich genus in primary forest was also *Camponotus* (17 species), but *Polyrhachis* (11 species) was the richest in secondary forest (S3 Fig.).

Arboreal nesting ants dominated the canopy habitat. Only 27 (21%) species were not recorded from nests and those species made up less than 1% of all collected foraging individuals from both plots. The majority of those species belonged to genera known to nest terrestrially, e.g. *Anochetus*, *Aphaenogaster*, *Lordomyrma*, *Odontomachus*, *Rhytidoponera* (collected usually as singleton workers and all limited to primary forest, S1 Table). However, most of the individuals of foragers with no nests in the plots consisted of other arboreal species, particularly *Tetraponera nitida* in secondary forest and *Crematogaster* cf. *major* in the primary forest plot, that were probably nesting either in the small trees that we did not sample (DBH < 5 cm) or foraged from trees outside of our plots.

**Fig 3:**
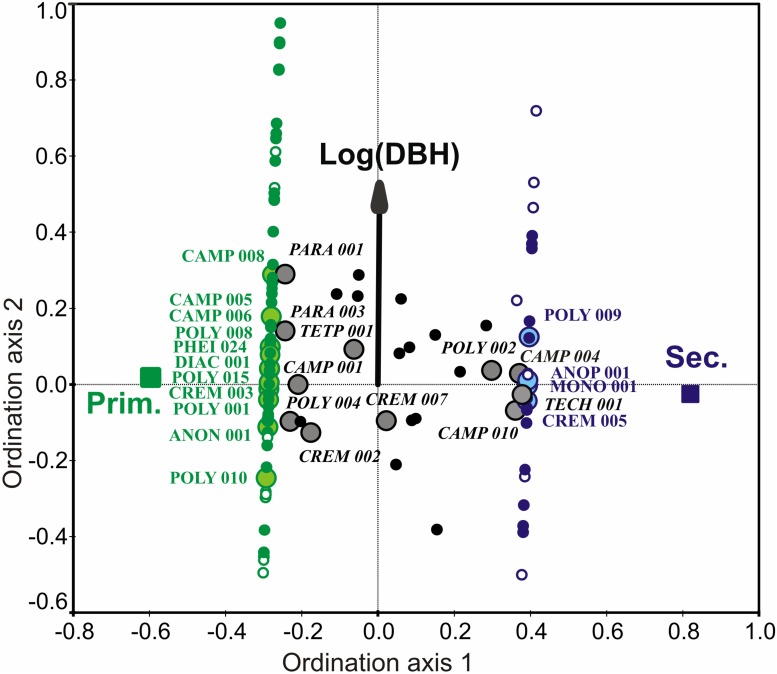
Ordination diagram of ant species composition in studied trees. Ordination based on CCA analysis (see Table 3 for the significance and % of variance related to the ordination canonical axes). Variation of ant community composition (all presence—absence records of ant species in trees) is related to the explanatory variables Forest plot on the first axis (primary and secondary rainforest) and Tree size on the second axis (DBH after logarithmic transformation). Solid symbols indicate tree-nesting species and empty symbols the species found only foraging in trees (in green: occurrence of the species in primary forest, in blue: secondary forest, in black: both forests). The enlarged symbols with species abbreviations refer to the most common ant species (i.e. present in > 20 trees, see S1 Table for their full names).

The difference in species composition between the two forest types was high, as there were only 26 shared species (half the species from the secondary forest). This difference was much more evident when the frequencies of the species in trees were considered: only one of the 26 common species (with ≥ 20 occupied trees) *Crematogaster* sp.7 aff. *fritzi* (CREM007) was similarly frequent in both plots (Figs. 3 and S4). The canonical analyses based on occurrences of all species, foraging species and nesting species all yielded very similar results (Table 3). Most of the variation in the ant communities in the CCA was explained by forest plot (successional stage) on the first canonical axis, followed by DBH on the second axis in all analysis. Only in the dataset on foraging ants was there some residual variance significantly explained by other tree size traits, i.e. tree height and crown width (Table 3). Difference in ant species composition between plots explained 2.5% to 9.9% of the variability in the nesting and foraging datasets respectively (CCA) and the proportion of total variability explained by the forest stage was very high (1st axis; mean CCA / CA = 81%). Tree traits generally explained a small proportion of the species variability compared to identity of forest (< 1% on 2nd CCA axis; max. CCA / CA = 21%; nest records). Species distribution along the tree size gradient was driven by rare ant species, while most of the common species were only weakly correlated with this gradient (Fig. 3). However, some common ant species preferred to nest in a tree of a particular size, especially in the arboreal genera *Camponotus* and *Polyrhachis* (e.g. the species CAMP008, POLY009 nested high in the canopies of large trees; POLY010, CAMP010 preferred small trees, Fig. 3).

**Fig 4:**
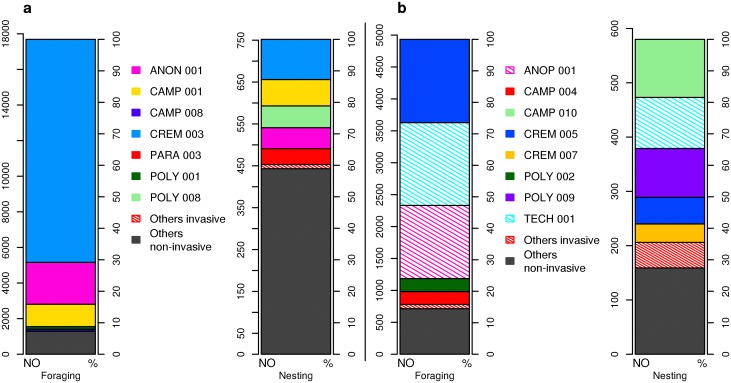
Abundance of tree-foraging and nesting ant species in each forest plot. Total individual abundance in % of number of foraging individuals (left column) and of arboreal nests (right column) collected in trees in primary (a) and secondary (b) forest plot. Different color patterns express the proportions of the five most abundant species and of the rest of the species respectively. Invasive species are cross-hatched (see S1 Table for full species names and their individual abundances).

The frequencies of ant species in trees expressed as the number of occupied trees as well as their abundances varied considerably between foraging and nesting ant communities (Figs. 4 and S4). For instance, the most common nesting species *Camponotus* cf. *macrocephalus* (CAMP010) occupied 88 trees but almost no foragers were observed. In contrast, *Anoplolepis gracilipes* was found to forage from the ground up into most of the secondary forest trees but its nesting incidence in trees was very low (Fig. 4, S1 Table) and only satellite nests were present. Generally, only a few species numerically dominated the foraging fauna in both plots, in contrast to the situation with nesting species (Fig. 4).

The primary forest community was characterized by *Crematogaster polita*, which accounted for the majority (71%) of all foraging individuals collected, and was also the most common nesting species (CREM003; Fig. 4). Although this species nested in only 13% of trees, it foraged on 64% of trees, and also monopolized the majority of canopy food resources, e.g. scale insects and extrafloral nectaries (S4 Fig., P. Klimes pers. observ.). Other dominant species in the community were *Anonychomyrma* cf. *scrutator* (ANON001) and *Camponotus vitreus* (CAMP001) (Figs. 4 and S4). Out of ten species considered to be invasive, six species were found in the primary forest plot among the foraging or nesting fauna (S1 Table). However, the overall abundance of invasive species collected in the primary forest plot was very small for both the number of foragers (0.01%) and nests (1.6%) (Fig. 4, S1 Table). For instance, only a single nest was found for each of the cosmopolitan invaders *Monomorium pharaonis* and *Tapinoma melanocephalum* there.

In contrast to the primary forest community, the most common species in the secondary forest were more evenly distributed in their abundance, for both foragers and nesters, while invasive ants were also common (Fig. 4, S1 Table; eight spp.). The secondary forest community was dominated by the invasive species *A*. *gracilipes* and *Technomyrmex brunneus* (TECH001), which made up 50% of foraging individuals collected (Fig. 4). Other invasive species in the secondary forest plot included e.g. *Monomorium floricola*, *Cardiocondyla obscurior*, and *Tapinoma melanocephalum* (S1 Table). The two most common native species in secondary forest, *Crematogaster flavitarsis* (CREM005) and *Polyrhachis neptunus* (POLY009), did not occur in the primary forest plot (Fig. 4, S1 Table).

### Models on nesting probabilities of tree-foraging ant communities

**Fig 5:**
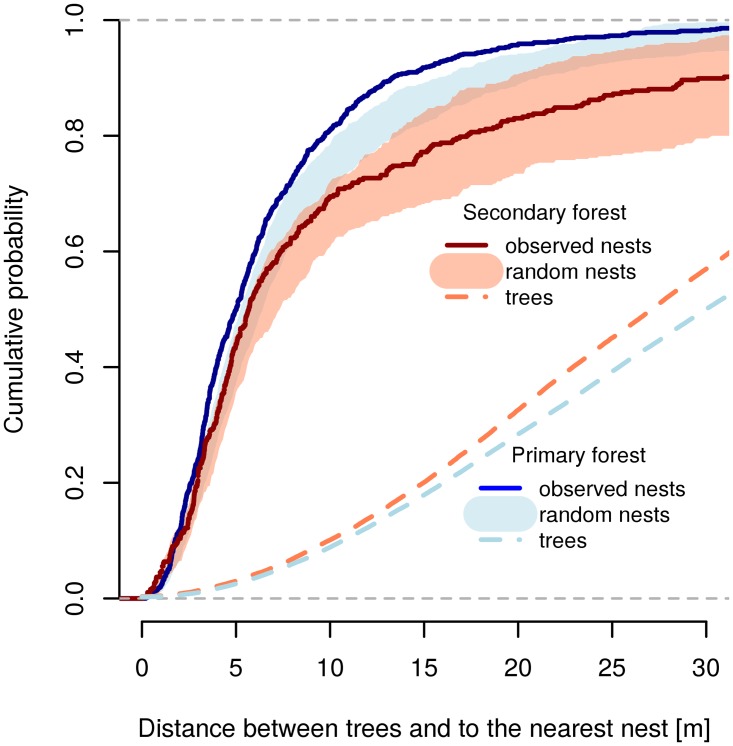
Cumulative probability of distances to the nearest nest of tree-foraging species. Curves based on observed nest data (F-N species records) for primary and secondary forest plots are compared to the area given by minimal and maximal values of 100 curves randomly generated by the permutations of the presence-absence matrix of nests in trees (see Methods for details and S3 Table for the data). Cumulative probability of distances between all trees in each forest plot is indicated by dashed lines.

Trees were closer to each other in secondary forest than in the primary forest plot (Fig. 5), with mean distance between all trees of 29.1 m (SD = 15.9) and 32.5 m (SD = 17.7), respectively. Despite this, the nearest nests for F-N ants were closer in primary forest than in secondary forest: 81% and 69% of F-N ants respectively had the nearest tree with a nest of that species less than 10 m from the tree where foragers were observed (Fig. 5). In the primary forest, the observed cumulative distribution of nearest nests was mostly above the randomly generated distribution (up to 25 m), and the observed mean distance (6.88 m) was significantly lower than the random mean (8.61 m; W = 0, p = 0.045). In the secondary forest, the observed distribution of the nearest nests did not differ significantly from the random distribution model (observed mean = 11.77 m; the random mean = 11.17 m; W = 80, p = 0.31).

**Fig 6:**
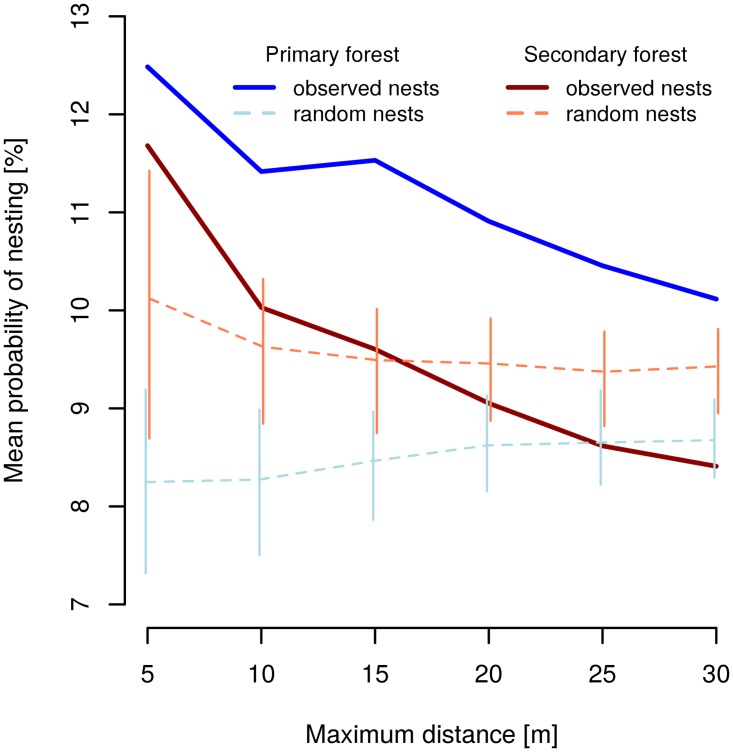
Probability of nesting of tree-foraging species in surrounding trees. Mean nesting probability of ant foragers (F-N species records) is calculated with increasing maximum distance from tree where they forage (but not nest) in the primary and secondary forest plot. Means are shown for observed data and 100 random permutations of the presence-absence matrix of nests, including 95% confidence interval envelopes (2.5% to 97.5 quantile range) of the model (see Methods and S1 Text for details on calculation).

The chances of finding F-N species in other trees decreased with increasing distance from the focal tree in both primary and secondary forest. On average, there was a 12.5% probability of a nest occurrence of F-N species in the primary forest and 11.7% in the secondary forest up to 5 m from the focal tree (Fig. 6). However, the effect of increasing distance was stronger in the primary forest plot, where the observed mean probability of nesting was always higher than the null model probability for all distances, and only 8.2% of trees up to 5 m were expected to have a nest of F-N species using randomized data (i.e. 1.5 times lower probability than observed). In contrast, in the secondary forest the observed mean probability of nesting was significantly higher that the randomly generated values only up to the 5 m scale (Fig. 6). However, a reversed difference was found for the furthest distances, where the observed values were significantly lower than those generated by random (i.e. F-N ant records were significantly more regularly distributed when considering distances within 25 m and more from the focal tree).

## Discussion

### Ant species richness and contribution of foragers to canopy ant fauna

The use of distribution data on trees from whole continuous forest plots has considerably advanced our understanding of tree species diversity and coexistence in the tropics [79,80]. To our best knowledge, our study is the first that presents such data for invertebrates living in trees, since collecting census data for numerous mobile organisms like insects is logistically challenging.

We estimated that in just two 0.32 ha plots of a lowland forest there are 140 and 62 different arboreal ant species coexisting in primary and secondary forest respectively. Although larger local ant species richness estimates have been reported before for tree canopies, for example over 400 spp. in 0.16 km^2^ in the canopy of a Neotropical forest [45], the previous studies sampled ants across much larger scales, which makes direct comparisons difficult. However, our results challenge the traditional view that a very high diversity of arboreal ant species exist on individual trees. Despite the high total (gamma) ant diversity, we found rather low mean species richness per tree (alpha diversity); 3.6 species. This contrasts with earlier studies which usually reported a higher alpha diversity, ranging from 4 to 53 spp. per tree [25,26,33,46,47]. We assume that the larger spatial scale and partially selective focus on large trees led to higher estimates of ant alpha diversity in previous studies. In this study, the inclusion of all trees with DBH over 5 cm significantly contributed to the overall low mean values as most trees in rainforests are small and hence host a low number of species (57% of the trees we sampled had DBH ≤ 10 cm and hosted only one ant nest on average). Similar findings of “empty trees” or trees with very low occurrence of ants when considering tree individuals of all sizes has also been found elsewhere [42,81] and this should be considered in future assessments of ant diversity in trees. Indeed, the differences in beta diversity and size among trees, as well as between successional stages, play important roles in structuring the diversity patterns of arboreal ant communities [22,82]. The scale of studies and selection of particular trees and sampling methods thus have important consequences for the overall estimation of species richness.

Ant species richness in primary forest was double that of secondary forest and this difference was proportionally similar whether considering foragers, nests, or both combined. This agrees with our prediction that species richness declines due to forest conversion and fragmentation [10,52,83,84]. However, mean overall species richness per tree only differed slightly between the two forests (3.3 in secondary and 3.8 in primary forest). This is surprising as previous studies usually found much larger differences in alpha diversity both for arboreal and litter ants between mature and disturbed forest habitats [52,85]. Furthermore, this difference was here only due to the higher foraging species density on the primary forest trees as the two forest plots did not differ in mean species richness per tree when only nests were considered. This suggests that the nesting and foraging spaces on individual trees are utilized similarly in both forest types despite the large differences in the structural characteristics of the vegetation between our primary and secondary forest plots [22,59]. The small difference in overall richness per tree between the two forests is also surprising due to the invasion of the secondary forest plot by a serious pest species *Anoplolepis gracilipes* [86]. This species has been shown to decrease species richness of arboreal ants in Indonesian agroforests [87]. However, this does not seem to be the case in our study despite the extraordinary ability of *A*. *gracilipes* to occupy 70–90% of canopy baits at the same site [76]. One plausible explanation for how relatively high numbers of species could still coexist with a dominant ant species like *A*. *gracilipes* in secondary forest is the utilization of their own canopy homopteran insect symbionts for honeydew near their nests [27,88]. Further studies focusing on the sampling of ants from whole tree communities including nests are needed to assess if the robustness of ant species richness to habitat degradation at the level of individual trees is common for tropical forests, as well as the role of inter-specific interactions (i.e. such as the presence of invasive species) in driving those patterns [18].

One crucial result from our study is the discovery of considerable variation in the diversity patterns within individual trees for foraging, nesting, and combined faunas. We assumed that ant communities occurring on individual trees are mosaics of true arboreal nesters and foraging visitors predominantly from the surrounding vegetation. Indeed, our study shows that the diversity of ants actually nesting on focal trees is considerably lower. In particular, only some nesters were actively foraging on their host trees, while other arboreal species foraged extensively also in trees in which they were not nesting. Interestingly, these patterns were very similar in the two forest types, despite the generally higher foraging species richness and ant activity in primary forest. Although numerous studies have reported the occurrence of many rare species in a tree, e.g. [15,16,47], they failed to quantify the contribution of ant foragers to ant canopy diversity. Floren [51] assessed ant nest diversity using direct observation of baits on branches before the trees were fogged. Similarly to our study, he found low diversity of ants coming from nests within a tree and concluded that “most of the numerous rare species probably entered the tree from neighbouring trees”, but did not provide nest data and quantitative comparison among forest types. Our study is the first to explicitly examine the foraging and nesting fauna separately and in such detail. Nevertheless, we are aware of the fact that the difference between foraging and nesting ants could be even higher as the richness of foraging species in our study could have been partly underestimated. In particular, collecting by hand could have led to the under-sampling of small and rare foraging species and we also did not search for nocturnal species foraging at night [33]. However, accumulation curves suggested that the sampling was representative for both the nesting and foraging fauna in our study.

Using the census data on foragers from whole forest plots and our null modelling approach we showed that foraging communities in trees are spatially dependent, with a significantly higher chance of encountering a nest of a foraging species not nesting in a focal tree in nearby trees. This effect was much stronger for primary forest than secondary forest ants, where the nearest nests of foragers were distributed randomly. However, when considering overall tree occupancy (i.e. nesting probability), this spatial relationship was observed in the secondary forest as well, although it was weaker and limited only to the closest trees. This result does not indicate that ants in secondary forest do not travel between tree canopies, as we found high foraging richness in trees in this habitat and there was no evidence that those species are not arboreal (S1 Table). In fact, there was a rather surprisingly low abundance of forest floor nesting ants in trees. This is in contrast to results from other geographic regions [25,33,89,90], although the low activity of ground-nesting ants in vegetation in PNG is in agreement with the observations of Wilson [91]. However, the important exception in our study is *A*. *gracilipes*, which prefers nesting on the ground but forages commonly up to the canopies [75,76,92].

There are several reasons why we did not discover a strong spatial pattern of foragers to their nests in secondary forest. First, secondary forest trees have considerably lower beta diversity of ants compared to primary forest [22]. Hence, it is not surprising that tracking a strong spatial relationship is difficult as the same common ants occur in most of the trees. Second, as primary forests have a greater load of epiphytes, large climbers and lianas that enhance canopy connectivity and provide ants with numerous foraging corridors [39,90,93], workers can likely travel at longer distances among trees there than in secondary forest. Third, as we only measured distances between trunks and not full three dimensional distances between nests in canopies, we might also have missed part of the spatial variability. Nevertheless, this would be expected to affect the strength of spatial dependence more strongly for primary forest trees, which are larger with wider canopies, and we did not observe weaker spatial patterns in primary forest. Last, as the secondary forest trees were more clumped (i.e. with shorter mean distance among trees), this could enhance foraging only between the closest trees. However, the finding that secondary foraging ants were more regularly distributed than expected by chance at longer distances from the tree where they only foraged is surprising. Such a pattern is usually attributed to environmental filtering or high intra-species competition [74,94]. It is possible that this was also the case here, as the dominant tree-nesting ants in secondary forest (*Technomyrmex brunneus* and *Crematogaster flavitarsis*) do not build large polydomous colonies extending many trees and hence might be more limited by between-colony competition or by the clumped distribution of trees (at longer distances), unlike in primary forest where the territorial species (*Crematogaster polita* and *Anonychomyrma* cf. *scrutator*) were dominant. Unfortunately, we could not conduct direct behavioral tests of worker avoidance between trees (colonies) to test this hypothesis.

### Composition of ant communities and the effects of environmental variables

The composition of subfamilies and genera at our site is congruent with other studies of arboreal ants across tropical equatorial forests [25,33,45,46,50]. One third of all genera belonged to the most diverse ant subfamily in the world, Myrmicinae, while Formicinae was ranked second in generic richness. The latter was dominated by two genera *Camponotus* and *Polyrhachis*, known to be hyper-diverse on trees [45,46], and being also the most species-rich genera in our study. Another arboreal genus *Crematogaster* was moderately species-rich but was also one of the most common ants encountered. In contrast, the most species-rich genera found previously in PNG lowland forests, *Pheidole* and *Strumigenys*, [95] were neither the most diverse nor common in our study. Nevertheless, this is not surprising as most species in these genera are known to be limited to the forest floor and leaf litter, though some are known to be arboreal [91,96]. Similarly, the low proportion of poneroid subfamilies found here corresponds with their preference for nesting in leaf litter and soil [96,97]. Indeed, this ant group was entirely absent from the secondary forest plot, where, in contrast to the primary forest plot, microhabitats such as epiphytes and aerial soil were scarce [22].

Most variation in ant species composition was due to rainforest disturbance, since forest type explained a larger amount of variation in species composition than size-related traits of trees. We found only 26 shared species (i.e. 25% of primary forest species) of which only one was relatively equally common, despite the two plots being separated by only ~1 km distance and the secondary forest site being surrounded by primary vegetation. Moreover, this pattern was comparable for both tree-nesting and foraging ants. Although, our results should be treated with caution as they are based on only one replication within each forest successional stage, given our extensive plot-based sampling across nearly 700 trees, we argue that these results are probably representative of the general differences between the primary and secondary forests in the area. The same dominant species were observed in other studies of lowland forests in PNG [40,76,98] and the arboreal ant fauna from other regions also usually showed considerable variation in species composition between undisturbed and altered forest habitats [52,83,96]. Notably, Floren *et al*. [82] found in Borneo a similar proportion of primary forest ant species in secondary forests of similar (and older) age as here.

Tree size has been found previously to be an important predictor of ant species community composition for some tropical tree species [81,99]. At our plots, tree size (i.e. DBH) was the best predictor of overall ant species richness and the number of nesting species [22]. As DBH was also found to be the most significant tree-size trait influencing species composition, regardless of whether considering foraging, nesting or the complete ant fauna, we recommend using this variable as a surrogate for tree size in future studies. Nevertheless, the overall effect of tree size on the composition of ant communities was rather minor, although a small proportion of species preferred a particular size of tree. This suggests a weak vertical stratification of the arboreal ant communities within the studied forests. Moreover, although distinct stratification is usually found between ground and canopy ants in the tropics [17,45], some of the species found nesting in trees here were observed nesting at ground-level in the forest as well (i.e. mostly soil-nesting species, S1 Table).

Our study demonstrates that data about species composition and abundance can vary greatly depending on which measurements of ant activity are used (i.e. foraging versus nesting; numbers of nests or foragers versus the number of occupied trees). Although our collection method cannot be used for the direct estimation of biomass or the total number of ant workers per tree (in contrast to fogging studies for externally tree-foraging species), the individual abundances of collected workers and nests is still a better reflection of the relative abundance of species on trees than the number of occupied trees (P. Klimes pers. observ., Klimes *et*.*al* 2011). In particular, our results suggest that some common species rarely forage (e.g. *Camponotus*, *Tetraponera*), perhaps because of their cryptic habits of nesting with their homopteran symbionts in hollow branches and myrmecophytic plants [88,100], while some other common species forage extensively, e.g *Crematogaster polita* and *Technomyrmex brunneus*.

Primary forest was largely dominated by *C*. *polita* and also partly by *Anonychomyrma* cf. *scrutator*. The genera *Crematogaster* and *Anonychomyrma* are also known to be predominant in North Australia which is closely related to the PNG fauna [101]. Our findings agree with other studies from the region that found *C*. *polita* to also be the most numerous species in primary rainforests [40,98]. This species builds large carton nests on the bark of trees, sometimes up to several meters wide, especially on large trees. The observed high ratio of trees with nests to the trees with foragers suggests a large territorial activity and perhaps the existence of a single polydomous colony on multiple trees in our plot [18,98]. The super-abundance of this species might also explain the higher abundance of foragers collected on primary forest trees compared to secondary forest trees, where this species was lacking. However, the higher tree-occupancy and foraging activity of ants in primary forest might also be explained by a greater diversity of nest sites, higher canopy connectivity, and presence of numerous lianas [20,22,93].

In contrast to the primary forest, the secondary forest was dominated by species that are widespread and common in other Australasian regions and are known to occur in disturbed or edge habitats [35,72]. Whether these invasive species were introduced to New Guinea is not clear as most of them (e.g. *Anoplolepis gracilipes*, *Monomorium floricola*, *Tapinoma melanocephalum*) have their putative origin in Oriental or Australasian regions [72,73,86,102]. In particular, the presence of *A*. *gracilipes* in inland New Guinea near pristine forests is worrying as this species builds huge super-colonies and is ranked as one of the most invasive animals in the world, inflicting a serious impact on its environment [86,87]. However, our data agree with findings that it is usually limited to disturbed habitats and avoids primary forests [75,87,103]. Interestingly, we also found *Technomyrmex brunneus* to be widespread in the secondary forest plot. In contrast, *Technomyrmex albipes* was found in primary forest [76] and is a widespread ant in the Pacific [72], but invasive elsewhere [42]. *T*. *brunneus* has been proposed as a separate species rather than a subspecies of *albipes*, and almost all of its records come from Asia [104]. Therefore, we cannot exclude the possibility that *T*. *brunneus* is an introduced species to PNG, unlike *T*. *albipes*. However, previous studies considered the latter species also as invasive in PNG [75].

Although the rarity of invasive species and tramp species in the primary forest plot is in full agreement with the prediction that native forest communities are more resistant to ant invasion than the disturbed communities [13,53], there are two alternative explanations of the community composition differences between our plots. First, we cannot exclude the possibility that the rare occurrences of some invasive species may reflect recent establishments of these species due the damage by felled trees, especially in the primary forest plot. Hence, although we did not find that the ant diversity and abundance were influenced by increasing disturbance during the course of felling, such (limited) effects on taxonomic composition in both plots are still possible. Second, variability in species composition might be partly caused by natural variation in the availability of particular nesting microhabitats and tree species, as secondary forest was also characterized by some common native species of ants not occurring in the primary plot. These species may prefer naturally disturbed parts of forests such as young successional vegetation regenerating in gaps after tree falls [82,105,106]. Further research and replicated sampling in other areas is needed to test these hypotheses for tropical canopy ants.

### Conclusions and perspectives

Using the first tropical forest-plot based data on arboreal ant communities we have demonstrated the high diversity of ants in a primary rainforest and its limited vulnerability to invasive ant species, regardless of whether tree-nesting species or foraging species are considered. In contrast, the secondary (disturbed) forest is considerably less species-diverse and characterized by a higher occurrence of invasive species. The size of trees is a significant predictor of ant community composition, but is less important than the effect of the successional forest stage. The greater part of the total species richness of ants in individual trees is attributed to foraging species than to ant communities nesting in those trees. Null models show that the ants foraging but not nesting in a tree are more likely to nest in nearby trees than would be expected at random, indicating an influx of ant foragers from surrounding vegetation. However, this pattern is stronger in primary forest, probably due to higher canopy connectivity, increased foraging activity and higher species turnover between trees in this successional stage compare to secondary forest. Our study demonstrates the significant overall contribution of foragers to arboreal ant diversity and the relevance of primary vegetation for the conservation of native ant communities. As primary forests are being increasingly threatened by human activities, other similarly complex studies replicated across more forest plots and including other arthropod taxa are needed to test whether the diversity patterns observed here for ants have general relevance for our understanding of the diversity of invertebrate canopy communities and their conservation in the tropics.

## Supporting Information

S1 Fig Proportions of ant species collected in trees exclusively foraging, nesting or the both. (PDF)

S2 Fig Distribution of ant subfamilies and their generic richness in the primary and secondary forest plot. (PDF)

S3 Fig Distribution of ant genera and their species richness in the primary and secondary forest plot. (PDF)

S4 Fig Frequency of the most common ant species in primary and secondary forest plot expressed as number of occupied trees. (PDF)

S1 Table List of all ant species and their numbers of occurrences in the two forest plots. (XLSX)

S2 Table Data matrices of ant nest and foraging occurrences in individual trees. (XLSX)

S3 Table Data matrix of minimum distances to nests of species only foraging in a tree. (XLSX)

S4 Table Data matrix of the observed mean nesting probabilities of ant foraging species in surrounding trees within a distance radius. (XLSX)

S1 Text Calculation of probability of nesting of ant foraging species in surrounding trees. (PDF)
