# Supplementary material for: Disentangling the Diversity of Arboreal Ant Communities in Tropical Forest Trees
Source: PLoS One. 2015 Feb 25;10(2):e0117853. doi: 10.1371/journal.pone.0117853 (PMC4340929; doi:10.1371/journal.pone.0117853)
Supplement: S1 Fig — (PDF) [file pone.0117853.s001.pdf]

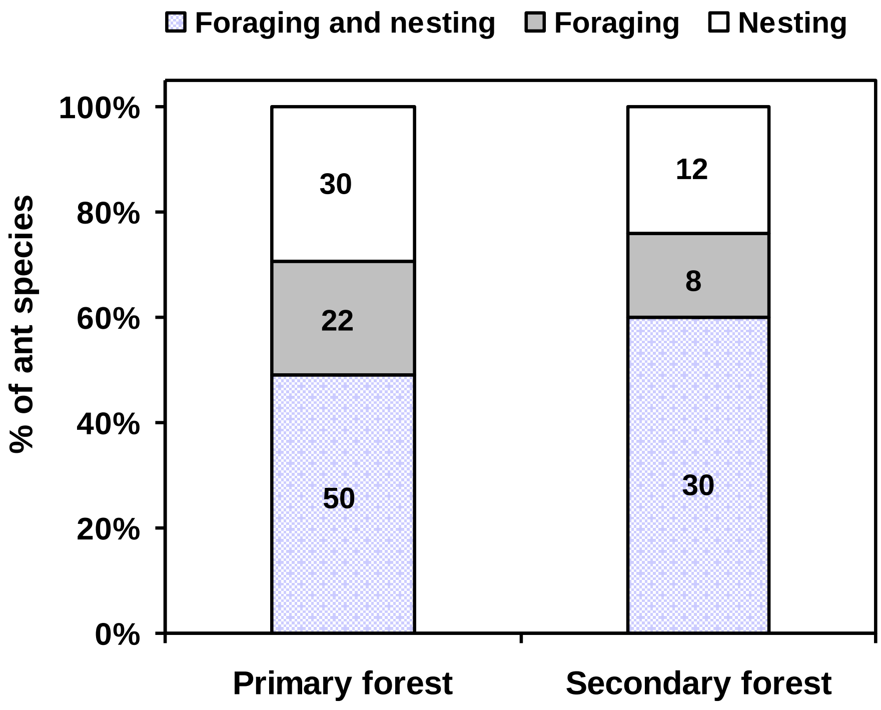

Figure S1. Proportions of ant species collected in trees exclusively foraging, nesting or the both. Numbers within columns refer to numbers of species in each category; frequencies of species distribution in categories do not differ between primary and secondary forest plots (Maximum-likelihood chi-square, $\chi^2 = 1.66$, d.f. = 2, $P = 0.43$).
