# Supplementary material for: Disentangling the Diversity of Arboreal Ant Communities in Tropical Forest Trees
Source: PLoS One. 2015 Feb 25;10(2):e0117853. doi: 10.1371/journal.pone.0117853 (PMC4340929; doi:10.1371/journal.pone.0117853)
Supplement: S2 Fig — (PDF) [file pone.0117853.s002.pdf]

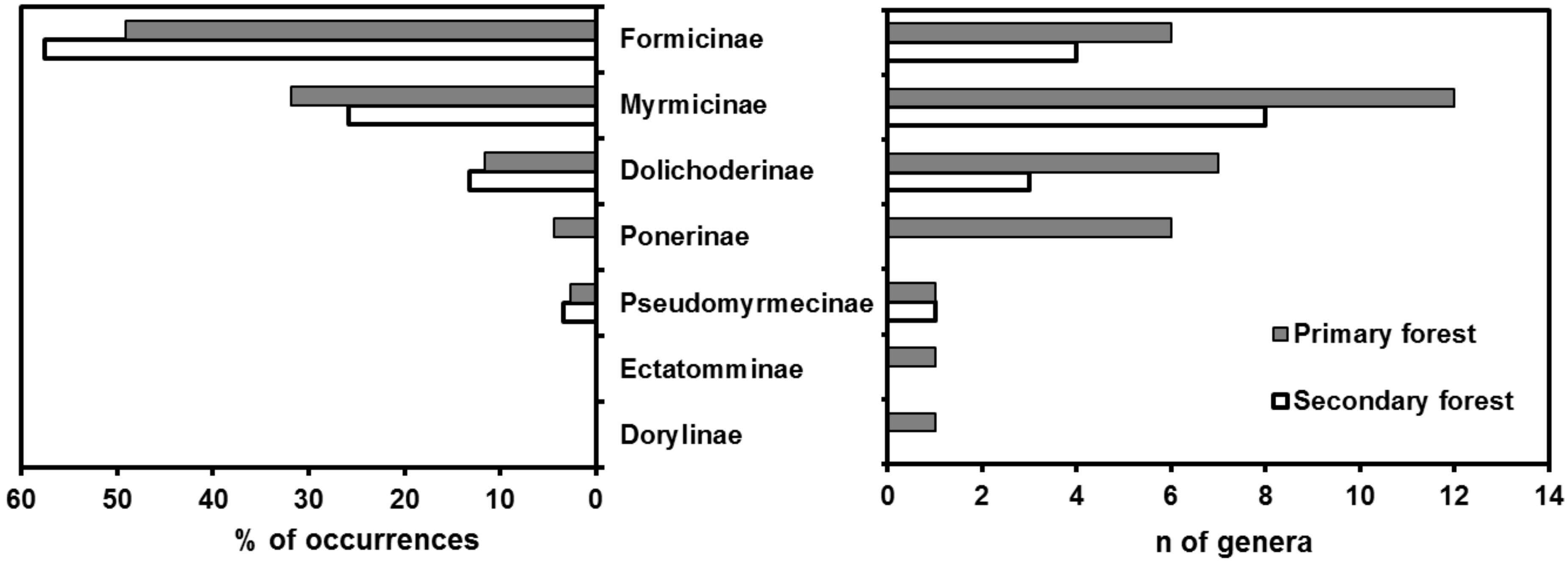

Figure S2. Distribution of ant subfamilies and their generic richness in the primary and secondary forest plot. Distribution of the subfamilies is expressed as their relative frequency in trees (percentage of total species occurrences in trees per forest plot) and their generic richness in primary and in secondary forest plot. All collected ant genera (n = 36) are included.
