# Supplementary material for: Disentangling the Diversity of Arboreal Ant Communities in Tropical Forest Trees
Source: PLoS One. 2015 Feb 25;10(2):e0117853. doi: 10.1371/journal.pone.0117853 (PMC4340929; doi:10.1371/journal.pone.0117853)
Supplement: S3 Fig — (PDF) [file pone.0117853.s003.pdf]

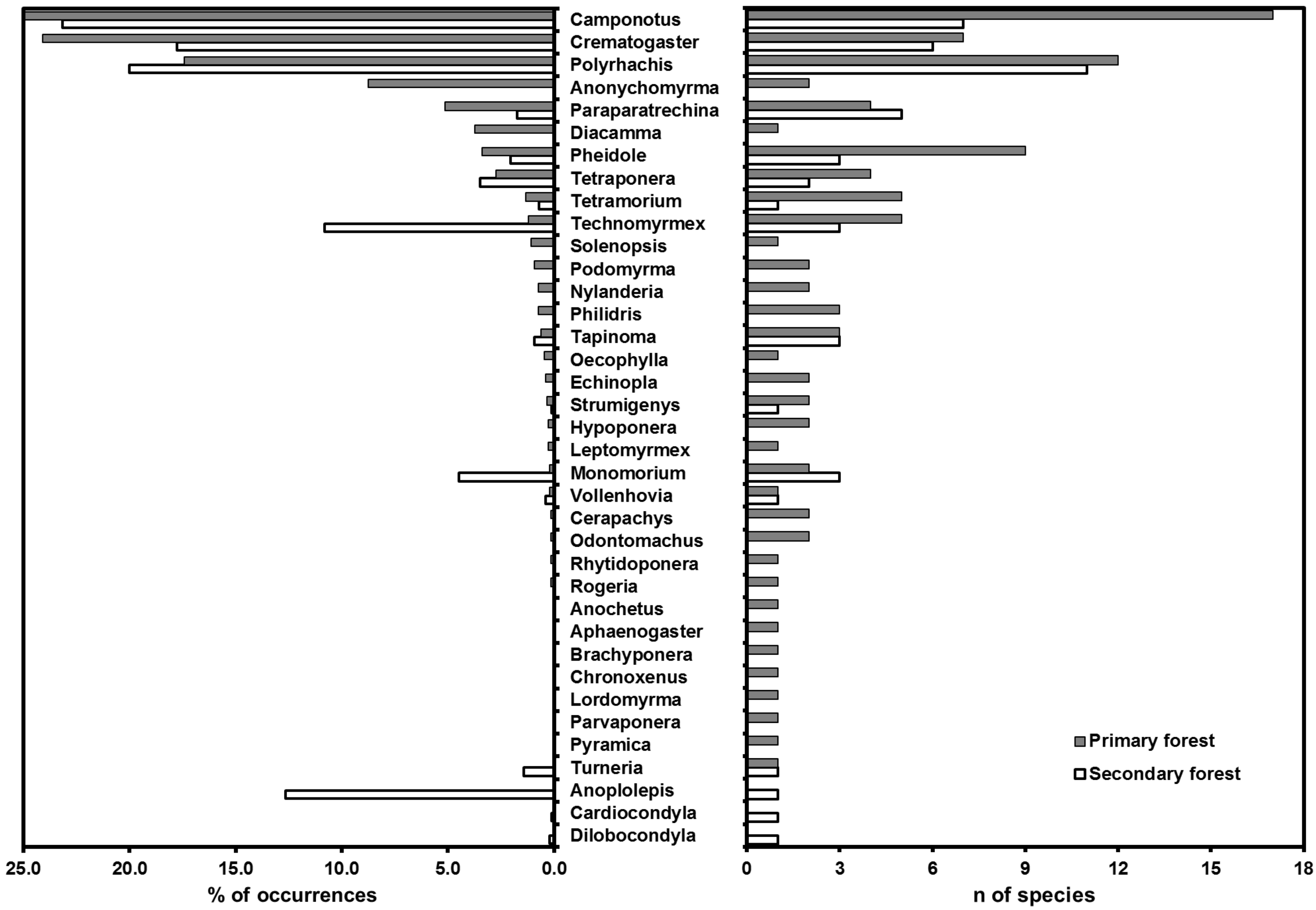

Figure S3. Distribution of ant genera and their species richness in the primary and secondary forest plot. Distribution of the genera is expressed as their relative frequency in trees (percentage of total species occurrences in trees per forest plot; left) and their species richness in primary and in secondary forest plot (right). All collected species (n = 126) are included and ordered by their % occurrence in primary forest plot.
