# Supplementary material for: Disentangling the Diversity of Arboreal Ant Communities in Tropical Forest Trees
Source: PLoS One. 2015 Feb 25;10(2):e0117853. doi: 10.1371/journal.pone.0117853 (PMC4340929; doi:10.1371/journal.pone.0117853)
Supplement: S4 Fig — (PDF) [file pone.0117853.s004.pdf]

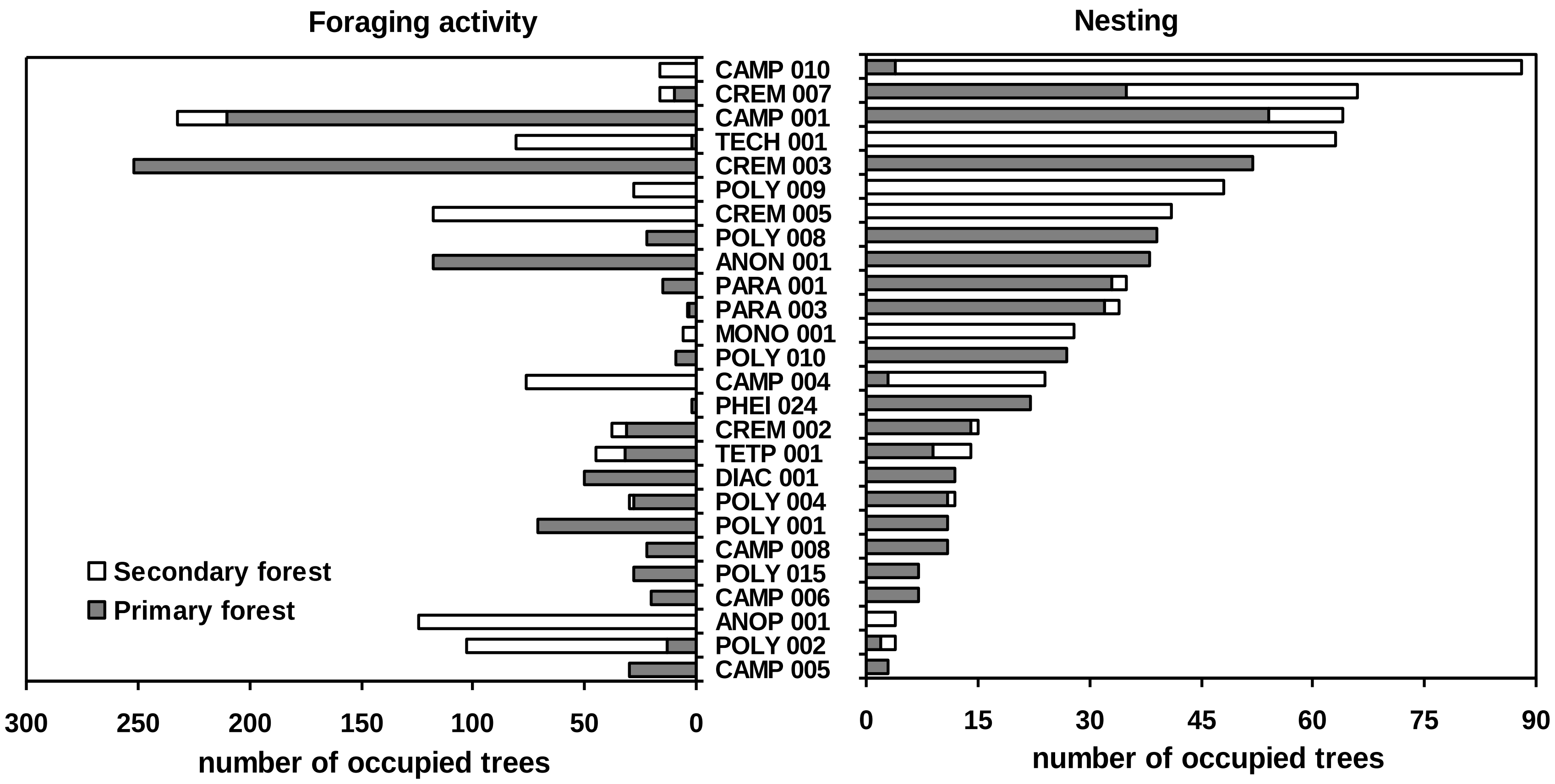

Figure S4. Frequency of the most common ant species in primary and secondary forest plot expressed as number of occupied trees. Relative frequency of the most common ant species in each forest plot (i.e. species present in > 20 trees, see Table S1 for full names) is expressed as number of trees with foragers (left) and number of trees with ant nests (right).
