# Supplementary material for: Disentangling the Diversity of Arboreal Ant Communities in Tropical Forest Trees
Source: PLoS One. 2015 Feb 25;10(2):e0117853. doi: 10.1371/journal.pone.0117853 (PMC4340929; doi:10.1371/journal.pone.0117853)
Supplement: S1 Text — (PDF) [file pone.0117853.s009.pdf]

Klimes P., Fibich P., Idigel C., Rimandai M. 2015. ***Disentangling the diversity of arboreal ant communities in tropical forest trees*** **(Supplementary material)**

Text S1

Calculation of probability of nesting of ant foraging species in surrounding trees

Method:

We defined S_t as a set of F-N ant species on the tree *t* (i.e. species foraging and not nesting on *t*), T_{td} as a set of all trees up to a given distance *d* in the plot from the tree *t*. We then computed the mean probability of nesting of F-N species on *t* up to distance *d* as a proportion of the number of observed nests from the number of all potential nests (trees) occurring up to *d* as:

$$100 \frac{\text{NO nests of species in } S_t \text{ on trees in } T_{td}}{\text{NO species in } S_t * \text{NO trees in } T_{td}}$$

where NO denotes the number of nests, ant species or trees. Probability of nesting is computed for each tree and each distance separately (trees times distances matrix, **Table S4**).

Example:

Assume that we are computing probability of nesting of foragers in other trees for tree *t* and distance *d*. Tree *t* has five F-N ant species (foraging but not nesting on *t*) denoted as A, B, C, D and E (set S_t). There are four trees up to the distance *d* from the tree *t* denoted as t1, t2, t3 and t4 (set T_{td}). Input matrix for the calculation contains data about presence/absence (1/0) of the nests of ant species in S_t on the trees in T_{td}

	**A**	**B**	**C**	**D**	**E**
**t1**	1	0	0	0	1
**t2**	1	1	1	1	0
**t3**	0	0	0	0	1
**t4**	0	1	1	0	0

Probability of nesting for the tree *t* is then 9 (sum of all presences in the matrix) divided by product of 5 (number of F-N species, S_t) and 4 (number of trees up to *d*, T_{td}) that together equals 0.45:

9 / (5 * 4) = 0.45

Hence, F-N species on *t* has mean probability 45% to nest on another tree find up to distance *d*.
